# Adaptive evaluation of gross total resection rates for endoscopic endonasal approach based on preoperative MRI morphological features of pituitary adenomas

**DOI:** 10.3389/fonc.2024.1481899

**Published:** 2024-12-17

**Authors:** Ao Shen, Yue Min, Dongjie Zhou, Lirui Dai, Liang Lyu, Wenyi Zhan, Shu Jiang, Peizhi Zhou

**Affiliations:** ^1^ Department of Neurosurgery, West China Hospital/West China School of Medicine, Sichuan University, Chengdu, Sichuan, China; ^2^ Department of Neurosurgery, West China Hospital/West China School of Nursing, Chengdu, Sichuan, China

**Keywords:** pituitary adenomas, anatomical landmarks, adaptive evaluation, prediction model, survival analysis

## Abstract

**Objective:**

This study aims to define a set of related anatomical landmarks based on preoperative Magnetic Resonance Imaging (MRI) of patients with pituitary adenomas (PAs). It explores the impact of the dynamic relationships between different anatomical landmarks and the tumor on the resection rate and tumor progression/recurrence during the endoscopic endonasal approach (EEA).

**Methods:**

A single-center institutional database review was conducted, identifying patients with PAs treated with EEA from December 2018 to January 2023. Clinical data were reviewed, and anatomical landmarks were categorized into two regions: the suprasellar region and the cavernous sinus region. Following basic statistical and univariate logistic regression analyses, patients were randomly divided into training and validation sets. A nomogram was then established through the integration of least absolute shrinkage and selection operator (LASSO) regression and multivariable logistic regression analysis. The clinical prediction model was evaluated using the area under the receiver operating characteristic curve (AUC), calibration curves, and decision curve analysis. Kaplan-Meier curves were plotted for survival analysis.

**Results:**

A total of 626 patients with PAs were included in the study, with gross total resection (GTR) achieved in 570 cases (91.05%). Significant differences were observed in the distribution of age, Knosp grade, and tumor size between the GTR and near total resection (NTR) groups. LASSO regression identified 8 key anatomical landmarks. The resulting model demonstrated an AUC of 0.96 in both the training and validation sets. Calibration curves indicated a strong agreement between the nomogram model and actual observations. Survival analysis revealed that the extent of resection (EOR), age, Knosp grade, tumor size, and PAs extending beyond several anatomical landmarks identified were significantly associated with the progression or recurrence of PAs.

**Conclusion:**

This study proposes a model for adaptively assessing the resection rate of PAs by delineating relevant anatomical landmarks. The model comprehensively considers instrument manipulation angles, surgical accessibility during EEA procedures, anatomical variations, and the displacement of related anatomical structures in pathological states. This approach can assist neurosurgeons in preoperative planning and developing personalized surgical strategies.

## 1 Introduction

Pituitary adenomas (PAs) are benign, slow-growing tumors that account for 10% to 25% of all intracranial tumors. These tumors can grow for extended periods without causing clinical symptoms. When symptoms such as vision loss, visual field defects, hypopituitarism, and headaches do appear, they typically indicate that the tumor has reached a significant size, resulting in mass effects. For functional adenomas, the initial symptoms are often endocrine syndromes caused by the overproduction of the corresponding hormones (1, 2).

The treatment of PAs varies depending on the type of adenoma. For prolactinomas (PRL adenomas), dopamine receptor agonists like bromocriptine are the first-line treatment. For other types of PAs, surgery is the preferred method (3–6). Historically, craniotomy and transsphenoidal microscopic surgery were widely used, with their advantages and disadvantages thoroughly discussed (7). With the advancement of endoscopic techniques, the endoscopic endonasal approach (EEA) has become increasingly utilized for the resection of almost all sellar lesions due to its superior visualization of the surgical field (8–11). The high resection rate and low complication rate of EEA have made it the preferred surgical technique for treating PAs.

Despite the benign nature of PAs, studies indicate that approximately 25% to 55% of these tumors exhibit aggressive growth behaviors. This is particularly evident in giant pituitary adenomas (GPAs), which often invade the cavernous sinus regions or encase critical vascular and neural structures, thereby affecting the resection rate (12, 13). Incomplete resection (NTR) of PAs can lead to adverse outcomes such as residual tumor hemorrhage and increased risk of recurrence. Therefore, even though the continuously optimized EEA technique has proven to be safe and effective, achieving maximal tumor removal, intratumoral decompression, and neural decompression without causing additional damage remains challenging in complex PAs (14–16).

Despite the various existing classification systems for assessing PAs invasions, they still have limitations. More precise and practical classification systems should be developed to better guide the surgical treatment of PAs, especially for complex cases involving cavernous sinus invasion and the suprasellar region. This study proposes a model for adaptively assessing PAs resection rates by delineating relevant anatomical landmarks. The model considers instrument manipulation angles, surgical accessibility during EEA procedures, anatomical variations, and displacement of structures in pathological states.

## 2 Materials and methods

### 2.1 Patient selection

**Figure 1:**
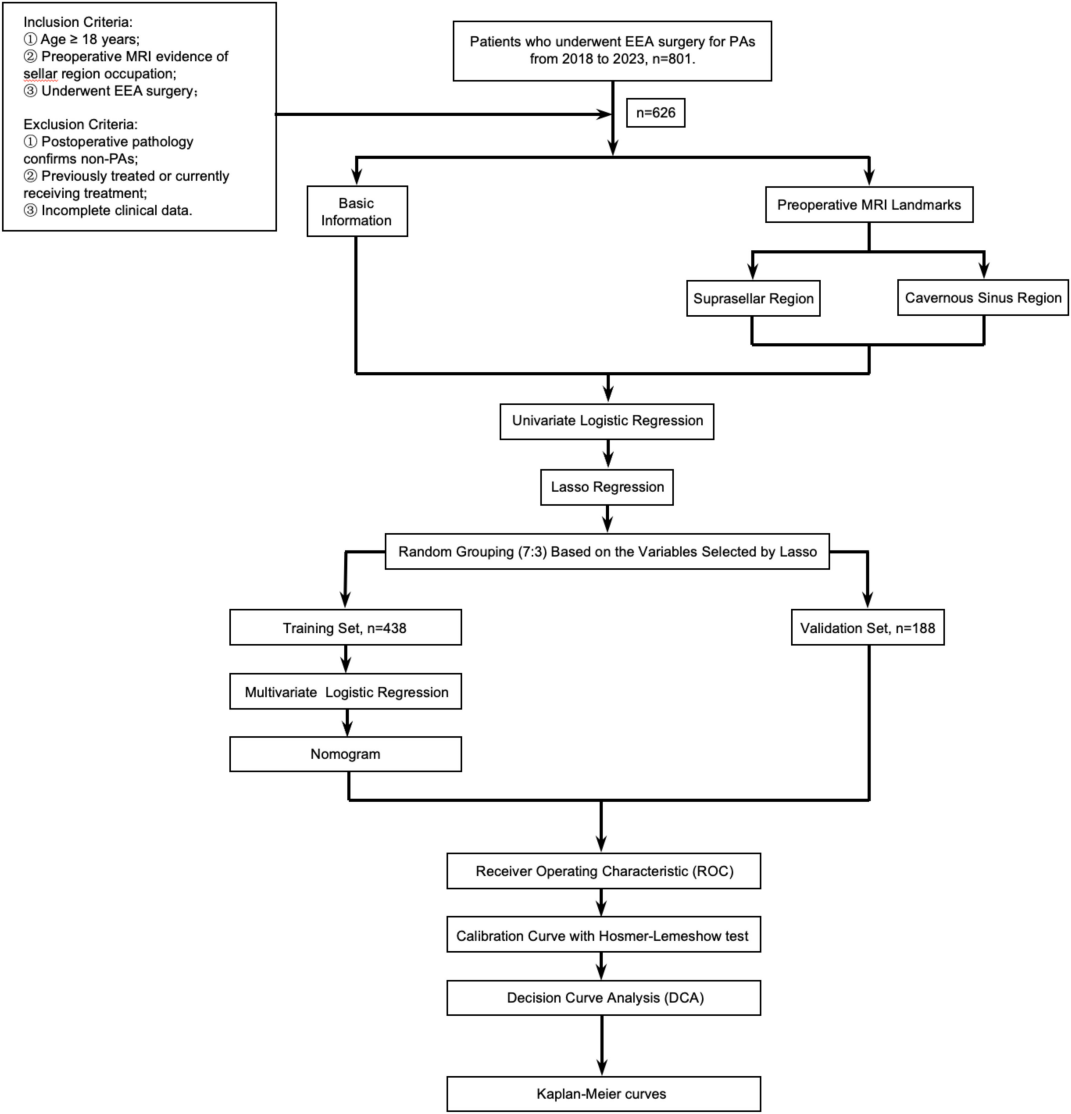
Flowchart of this study.

A retrospective review was conducted following IRB approval of our institution. It was conducted and reported in line with the STROBE criteria. We reviewed a collected database of all EEA surgeries performed by the senior author between December 2018 and January 2023. Inclusion criteria: 1) Age ≥ 18 years; 2) Preoperative enhanced MRI of the sellar region confirming a sellar mass; 3) Patients who underwent EEA surgery following the diagnosis of the sellar mass. Exclusion criteria: 1) Postoperative pathology confirmed non-PAs; 2) Patients who had previously undergone craniotomy, EEA surgery, or Gamma Knife treatment; 3) Cases with important clinical data missing, such as those without pre-treatment pituitary hormone assessment or incomplete medical records. 626 patients with PAs were ultimately included in the study. Data including patient demographics, baseline endocrinopathies, tumor characteristics (including tumor size, cavernous sinus (CS) invasion, suprasellar extension, and pathology), and operative outcomes (including EOR and recurrence/progression) (**Figure 1**).

### 2.2 Definition of anatomic landmarks

**Figure 2:**
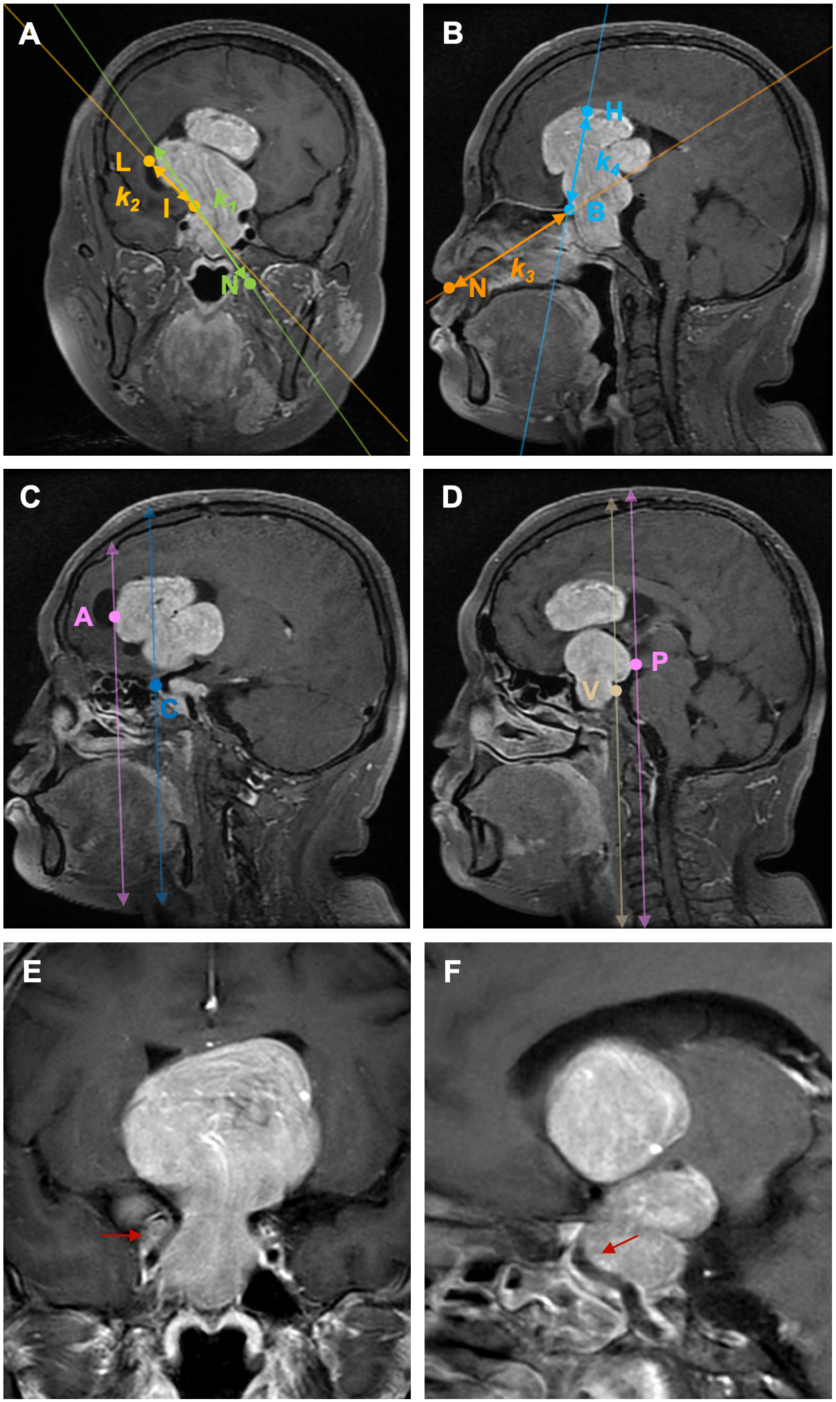
Illustration of the definition of each anatomical landmark on preoperative MRI. A: On coronal MRI, PAs extending laterally beyond the anatomical landmark in the suprasellar region is defined by |*k*_1_|of *l*_1_> |*k*_2_|of *l*_2_. B: On sagittal MRI, PAs extending superiorly beyond the anatomical landmark in the suprasellar region is defined by |*k*_3_|of *l*_3_> |*k*_4_|of *l*_4_. C: On sagittal MRI, PAs extending anteriorly beyond the anatomical landmark is defined as when the anteriormost point of the ICA bending **(C)** is posterior to the anteriormost horizontal extension point of PAs **(A)** on the same plane; **(D)** PAs extending posteriorly beyond the anatomical landmark is defined as when the peak point of the clivus (V) on the midsagittal MRI plane is posterior to the furthest horizontal extension point (P) of PAs on the same plane. **(E)** PAs invade the lateral ICA compartment of the CS; **(F)** PAs invade the posterior ICA compartment of the CS. PAs: pituitary adenomas, ICA: internal carotid artery; L: the furthest lateral horizontal extension point of the PAs, I: the clinoid segment of ICA, N: the projected point on the side of the nostril’s outer edge, **(B)** the furthest point of PAs extension towards the anterior skull base (sphenoid/ethmoid bone, or the boundary between the tumor tissue and normal brain tissue), H: the highest point of PAs extension above the saddle, A: the anteriormost horizontal extension point of PAs, C: the anteriormost point of the ICA bending, V: the peak point of the clivus, P: the furthest horizontal extension point of PAs.

**Figure 3:**
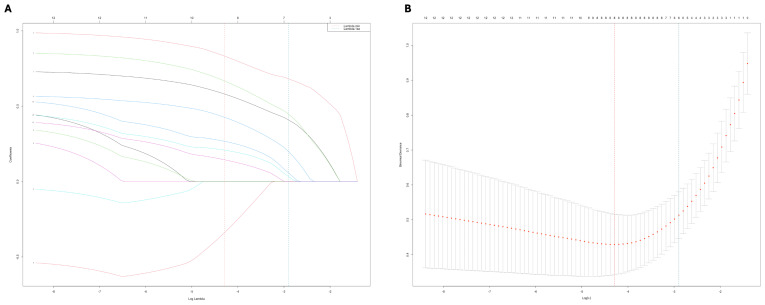
**(A)** LASSO coefficient profiles of 12 anatomical landmarks. Two vertical lines were added to indicate the λ values selected through fivefold cross-validation. Within this optimal λ range, 8 key anatomical landmarks with nonzero coefficients were identified. **(B)** The LASSO cross-validation plot displays the binomial deviance against the logarithm of the λ values. The optimal λ values are indicated by the vertical dashed lines: λ.min = 0.0094 and λ.1se = 0.0347. The λ.min value corresponds to the minimum mean cross-validated error, while λ.1se represents the most regularized model with a cross-validated error within one standard error of the minimum.

According to preoperative enhanced MRI of the sellar region, tumor size was defined as the maximal diameter in either the anteroposterior, lateral, or rostrocaudal dimension. Anatomical landmarks and anatomical areas were defined: 1) On coronal MRI, at the level where both the cavernous and clinoid segments of the internal carotid artery (ICA) are visible, a hypothetical line (*l*_1_) is defined by the furthest lateral horizontal extension point (L) of the PAs in the suprasellar direction and the clinoid segment of ICA (I) on the same side, with a defined slope of *k*_1_; 2) On coronal MRI, the projected point (N) on the side of the nostril’s outer edge, at the level where the cavernous and clinoid segments of the ICA opposite to the PAs’ suprasellar lateral extension are visible, and the clinoid segment of the ICA (I) at the same level, a hypothetical line (*l*_2_) is established with a defined slope of *k*_2_.PAs extending laterally beyond the anatomical landmark in the suprasellar region is defined by |*k*_1_|of *l*_1_> |*k*_2_|of *l*_2_ (**Figure 2A**); 3) On sagittal MRI, the furthest point of PAs extension towards the anterior skull base (sphenoid/ethmoid bone, or the boundary between the tumor tissue and normal brain tissue) (B), and the highest point of PAs extension above the saddle (H) on the same level, a hypothetical line *l*_3_ is determined with a slope of *k*_3_; 4) A hypothetical line *l*_4_ is established between point B and the projection point (N) of the nostril on the same side in the sagittal position, each with defined slopes of *k*_4_. PAs extending superiorly beyond the anatomical landmark in the suprasellar region is defined by |*k*_3_|of *l*_3_> |*k*_4_|of *l*_4_ (**Figure 2B**); 5) On the sagittal MRI where the anterior bending of one side ICA is clearly visible, when the anteriormost point of the ICA bending (C) is posterior to the anteriormost horizontal extension point of PAs (A) on the same plane, it is considered that PAs are extending anteriorly beyond the anatomical landmark (**Figure 2C**); 6) PAs extending posteriorly beyond the anatomical landmark is defined as when the peak point of the clivus (V) on the midsagittal MRI plane is posterior to the furthest horizontal extension point (P) of PAs on the same plane (**Figure 2D**); 7) The lateral and posterior regions of the cavernous sinus segment of the ICA as defined by Juan C. Fernandez-Miranda (17) (**Figures 2E, F**): The lateral compartment of the CS lies lateral to the anterior genu and horizontal segments of the ICA. Its upper boundary is the proximal dural ring covering the optic strut, and its lower boundary is defined by the maxillary strut and the V2 prominence. The anterior limit is marked by the point where the cranial nerves enter the superior orbital fissure and exit the cavernous sinus. The posterior compartment of the CS is located posterior to the short vertical segment of the ICA and anterior to the lateral petroclival dura, forming the posterior wall of the CS; Based on the side and location of ICA involvement by PAs, 8 subgroups were defined: unilateral, bilateral, uniposterior, biposterior, unilateral + uniposterior, unilateral + biposterior, bilateral + uniposterior, bilateral + biposterior CS invasions (**Table 1**).

**Table 1:** The definition of anatomical landmarks.

Regions	Anatomical landmarks			Definition	
Suprasellar	Lateral	Coronal MRI: both the cavernous and clinoid segments of the ICA are visible	L: The farthest lateral horizontal extension point of PAs in the suprasellar region.	$k_{1}=\frac{y_{L}-y_{N}}{x_{L}-x_{N}}$ $k_{2}=\frac{y_{I}-y_{N}}{x_{I}-x_{N}}$	$\left|k_{1}|>|k_{2}\right|$ lateral extension exceeds the landmark.
			N: The projected point of the nostril at this level.		
			I: The clinoid segment of the ICA (I) at the same side as the lateral extension.		
	Superior	Midsagittal MRI	B: The farthest point of PAs extension towards the anterior skull base (sphenoid/ethmoid bone)/the boundary between PAs and normal brain tissue at the anterior skull base.	$k_{3}=\frac{y_{B}-y_{N}}{x_{B}-x_{N}}$ $k_{4}=\frac{y_{H}-y_{N}}{x_{H}-x_{N}}$	$\left|k_{3}|>|k_{4}\right|$ superior extension exceeds the landmark.
			H: The highest point of PAs extension.		
			N: The projected point of the nostril at this level.		
	Anterior	Sagittal MRI: the anterior bend of one side of the ICA is clearly visible	A: The farthest anterior horizontal extension.	A > C: anterior extension exceeds the landmark.	
			C: The most anterior point of the ICA bend.		
	Posterior	Midsagittal MRI	P: The farthest posterior horizontal extension point.	P > V: posterior extension exceeds the landmark.	
			V: The clivus peak.		
Cavernous Sinus	Lateral	Unilateral			
		Unilateral + uniposterior			
		Unilateral + biposterior			
		Bilateral			
		Bnilateral + uniposterior			
		Bnilateral + biposterior			
	Posterior	Uniposterior			
		Biposterior			

PAs, pituitary adenomas; ICA, internal carotid artery; MRI, Magnetic Resonance Imaging.

### 2.3 Endocrinological evaluation

A comprehensive pituitary panel was obtained preoperatively, including measurements of prolactin (PRL), thyroid-stimulating hormone (TSH), free triiodothyronine (FT3), free thyroxine (FT4), adrenocorticotropic hormone (ACTH), random cortisol, follicle-stimulating hormone (FSH), luteinizing hormone (LH), growth hormone (GH), and insulin-like growth factor-1 (IGF-1). According to clinical subtypes, PAs were classified into non-functioning pituitary adenomas (NFPAs), PRL adenomas, GH adenomas, ACTH adenomas, TSH adenomas, and mixed adenomas. Follow-up endocrinological testing was regularly performed upon discharge and 3 months postoperatively to assess for biochemical remission in functional adenomas and the improvement or worsening of hypopituitarism.

### 2.4 Follow-up

All included patients were followed up for 22-59 months. The recurrence of PAs is defined as the appearance of new lesions at the original surgical site observed on imaging. For functional adenomas, a rise in hormone levels accompanied by imaging evidence should be considered. Gross total resection (GTR) was defined as no residual tumor on MRI at 3 months, and near total resection (NTR) was defined as residual tumor volume less than 10%. Recurrence of PAs was defined as evidence of a tumor mass on standard pituitary MRI scan during follow-up after previous GTR. Tumor progression was defined as evidence of regrowth of residual on MRI.

### 2.5 Statistical analysis

All data were entered and organized using Microsoft Excel (Version 16.84). Statistical analyses and data visualization were completed using R in RStudio (Version 2023.06.0 + 421). The Kolmogorov-Smirnov test was employed to determine if continuous variables followed a normal distribution. For normally distributed continuous variables, the mean ± standard deviation was used to describe central tendency and dispersion. The independent samples t-test was used to compare two independent samples for normally distributed continuous variables. The Chi-square test was applied to examine the correlation between two or more categorical variables. For small datasets or when the expected frequency in any cell was less than 5, Fisher’s exact test was utilized. Univariate logistic regression was conducted for univariate risk assessment. Patients were randomly assigned to training and validation sets in a 7:3 ratio. LASSO regression was used to address multicollinearity and variable selection. Receiver Operating Characteristic (ROC) curves were plotted to evaluate the multivariate logistic regression model based on dichotomous variables. The area under the ROC curve (AUC) measured the model’s performance in correctly distinguishing between positive and negative classes. Nomograms were plotted for quantitative risk assessment, and clinical decision curves were employed to evaluate the net benefit of using the model for prediction at different threshold probabilities compared to not using the model. Kaplan-Meier curves were plotted to describe the changes in tumor recurrence/progression over time in different groups.

## 3 Results

### 3.1 Patient demographics and clinical characteristics

626 patients were analyzed based on the extent of resection (EOR). 331 (52.88%) were male and 275 (47.12%) were female. Patients under 60 years old comprised 544 (86.90%) of the total, with 478 (87.87%) in the GTR group. Those aged 60 and above comprised 82 (13.10%) of the total, with 43 (52.44%) achieving GTR, showing a significant difference, with older patients being more prevalent in the NTR group (P < 0.05). The distribution of adenoma types was as follows: NFPAs in 529 (84.50%), PRL adenomas in 33 (5.27%), GH adenomas in 20 (3.19%), ACTH adenomas in 17 (2.72%), TSH adenomas in 8 (1.28%), and mixed adenomas in 19 (3.04%) patients. The Knosp grade distribution showed 86 (13.74%) patients in Grade 0, 183 (29.23%) in Grade 1, 205 (32.75%) in Grade 2, 61 (9.74%) in Grade 3A, 28 (4.47%) in Grade 3B, and 63 (10.06%) in Grade 4. A significant difference, with higher grades more common in the NTR group, was observed (P < 0.05). Additionally, significant differences between the GTR and NTR groups were found among the microadenomas (1.44%), macroadenomas (86.74%), and GPAs (11.82%) (P < 0.05) (**Table 2**).

**Table 2:** Patient demographics and clinical characteristics by EOR.

Variables, n (%)	Total (626, 100)	GTR (570, 91.05)	NTR (56, 8.95)	*P*
Gender, n (%)				
Male	331 (52.88)	247 (80.98)	58 (19.01)	
Femal	295 (47.12)	226 (82.18)	49 (17.82)	
Age, n (%)				<.001*
< 60	544 (86.90)	478 (87.87)	62 (12.20)	
≥ 60	82 (13.10)	43 (52.44)	39 (47.56)	
Clinical subtype, n (%)				0.713
NFPAs	529 (84.50)	479 (90.55)	50 (9.45)	
PRL adenomas	33 (5.27)	31 (93.94)	2 (6.06)	
GH adenomas	20 (3.19)	20 (100.00)	0 (0.00)	
ACTH adenomas	17 (2.72)	15 (88.24)	2 (11.76)	
TSH adenomas	8 (1.28)	8 (100.00)	0 (0.00)	
Mixed adenomas	19 (3.04)	17 (89.47)	2 (10.53)	
Knosp grade, n (%)				<.001*
0	86 (13.74)	86 (100.00)	0 (0.00)	
1	183 (29.23)	181 (98.91)	2 (1.09)	
2	205 (32.75)	191 (93.17)	14 (6.83)	
3A	61 (9.74)	42 (68.85)	19 (31.15)	
3B	28 (4.47)	20 (71.43)	8 (28.57)	
4	63 (10.06)	50 (79.37)	13 (20.63)	
Size, n (%)				<.001*
Microadenomas	9 (1.44)	9 (100.00)	0 (0.00)	
Macroadenomas	543 (86.74)	503 (92.63)	40 (7.37)	
GPAs	74 (11.82)	58 (78.38)	16 (21.62)	
Ki67, n (%)				0.197
< 3%	428 (68.37)	394 (92.06)	34 (7.94)	
≥ 3%	198 (31.63)	60 (88.89)	22 (11.11)	

GTR, Gross total resection; NTR, Near total resection; NFPAs, Non-functioning pituitary adenomas; PRL, Prolactin; GH, Growth hormone; ACTH, Adrenocorticotropic hormone; TSH, Thyroid-stimulating hormone; GPAs, Giant pituitary adenomas.The bold values and the symbol * indicate that the results are statistically significant.

### 3.2 Anatomical landmarks and surgery outcomes

For PAs’ suprasellar lateral extension, 123 (19.65%) patients were beyond the range (|*k*_1_| > |*k*_2_|), with only 75 (60.65%) achieving GTR. For suprasellar superior extension, 122 (19.49%) were beyond the range (|*k*_3_| > |*k*_4_|), and 74 (60.66%) achieved GTR. In terms of anterior extension, 75 (11.98%) were beyond the range (A > C), 45 (60.00%) achieving GTR. For posterior extension, 54 (8.63%) were beyond the range (P > V), and 30 (55.56%) achieved GTR. There were statistical differences between the EOR groups regarding whether PAs extended beyond the aforementioned four scenarios (P < 0.05). Regarding CS invasion, unilateral, bilateral, uniposterior and biposterior invasion were seen in 78 (12.46%), 128 (20.45%), 76 (12.14%) and 92 (14.70%), respectively. In the aforementioned four cavernous sinus regions, there were statistical differences between the two groups regarding whether PAs invaded (P < 0.05). For the combination of unilateral + uniposterior, unilateral + biposterior, bilateral + uniposterior, bilateral + biposterior involvement, the number of patients was 76 (12.14%), 43 (6.87%), 39 (6.23%), 31 (4.95%), respectively, significant differences were observed between the EOR groups in all conditions (P < 0.05) (**Table 3**).

**Table 3:** Anatomical landmarks and surgery outcomes by EOR.

Landmarks, n (%)		Total (626, 100)	GTR (570, 91.05)	NTR (56, 8.95)	*P*
Suprasellar	Lateral extension, n (%)				<.001*
	Witdin	503 (80.35)	495 (98.41)	8 (1.59)	
	Beyond $\left(\left|k_{1}|>|k_{2}\right|\right)$	123 (19.65)	75 (60.98)	48 (39.02)	
	Superior extension, n (%)22				<0.001*
	Witdin	504 (80.51)	490 (98.41)	8 (1.59)	
	Beyond $\left(\left|k_{3}|>|k_{4}\right|\right)$	122 (19.49)	74 (60.66)	48 (39.34)	
	Anterior extension, n (%)				<0.001*
	Witdin	551 (88.02)	525 (95.28)	26 (4.72)	
	Beyond (A > C)	75 (11.98)	45 (60.00)	22 (40.00)	
	Posterior extension, n (%)				<0.001*
	Witdin	572 (91.37)	540 (94.41)	32 (5.59)	
	Beyond (P > V)	54 (8.63)	30 (55.56)	24 (44.44)	
CS	Unilateral, n (%)				0.011*
	Non-invasion	548 (87.54)	493 (89.96)	55 (10.04)	
	Invasion	78 (12.46)	77 (98.72)	1 (1.28)	
	Bilateral, n (%)				<0.001*
	Non-invasion	498 (79.55)	442 (88.76)	56 (11.24)	
	Invasion	128 (20.45)	128 (100.00)	0 (0.00)	
	Uniposterior, n (%)				0.004*
	Non-invasion	550 (87.86)	481 (90.07)	53 (9.93)	
	Invasion	76 (12.14)	76 (100.00)	0 (0.00)	
	Biposterior, n (%)				0.039*
	Non-invasion	534 (85.30)	481 (90.07)	53 (9.93)	
	Invasion	92 (14.70)	89 (96.74)	3 (3.26)	
	Unilateral + uniposterior, n (%)				0.013*
	Non-invasion	550 (87.86)	495 (90.00)	52 (17.36)	
	Invasion	76 (12.14)	75 (98.68)	1 (1.32)	
	Unilateral + biposterior, n (%)				0.010*
	Non-invasion	583 (93.13)	536 (91.94)	47 (8.06)	
	Invasion	43 (6.87)	75 (98.68)	1 (1.32)	
	Bilateral + uniposterior, n (%)				<0.001*
	Non-invasion	587 (93.77)	544 (92.67)	43 (7.33)	
	Invasion	39 (6.23)	26 (66.67)	13 (33.33)	
	Bilateral + biposterior, n (%)				<0.001*
	Non-invasion	595 (95.05)	567 (95.29)	28 (4.71)	
	Invasion	31 (4.95)	3 (9.68)	28 (90.32)	

CS, Cavernous sinus; GTR, Gross total resection; NTR, Near total resection.The bold values and the symbol * indicate that the results are statistically significant.

### 3.3 Univariate logistic regression and LASSO regression analyses based on anatomical landmarks

**Figure 4:**
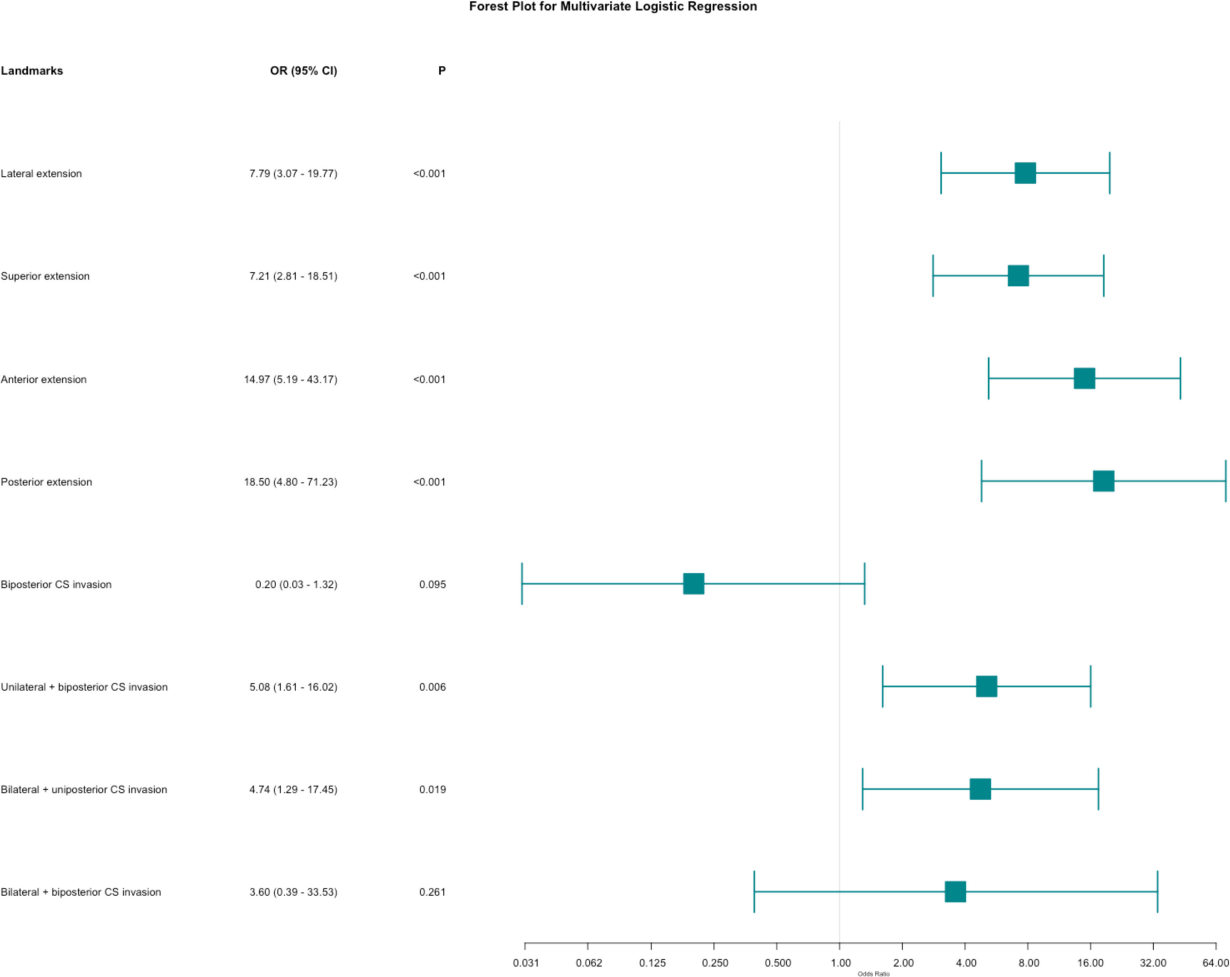
Forest plot for multivariate logistic regression for anatomical landmarks. The plot shows the odds ratios (OR) with 95% confidence intervals (CI) for various anatomical landmarks in relation to the likelihood of near-total resection (NTR) versus gross total resection (GTR).

**Figure 5:**
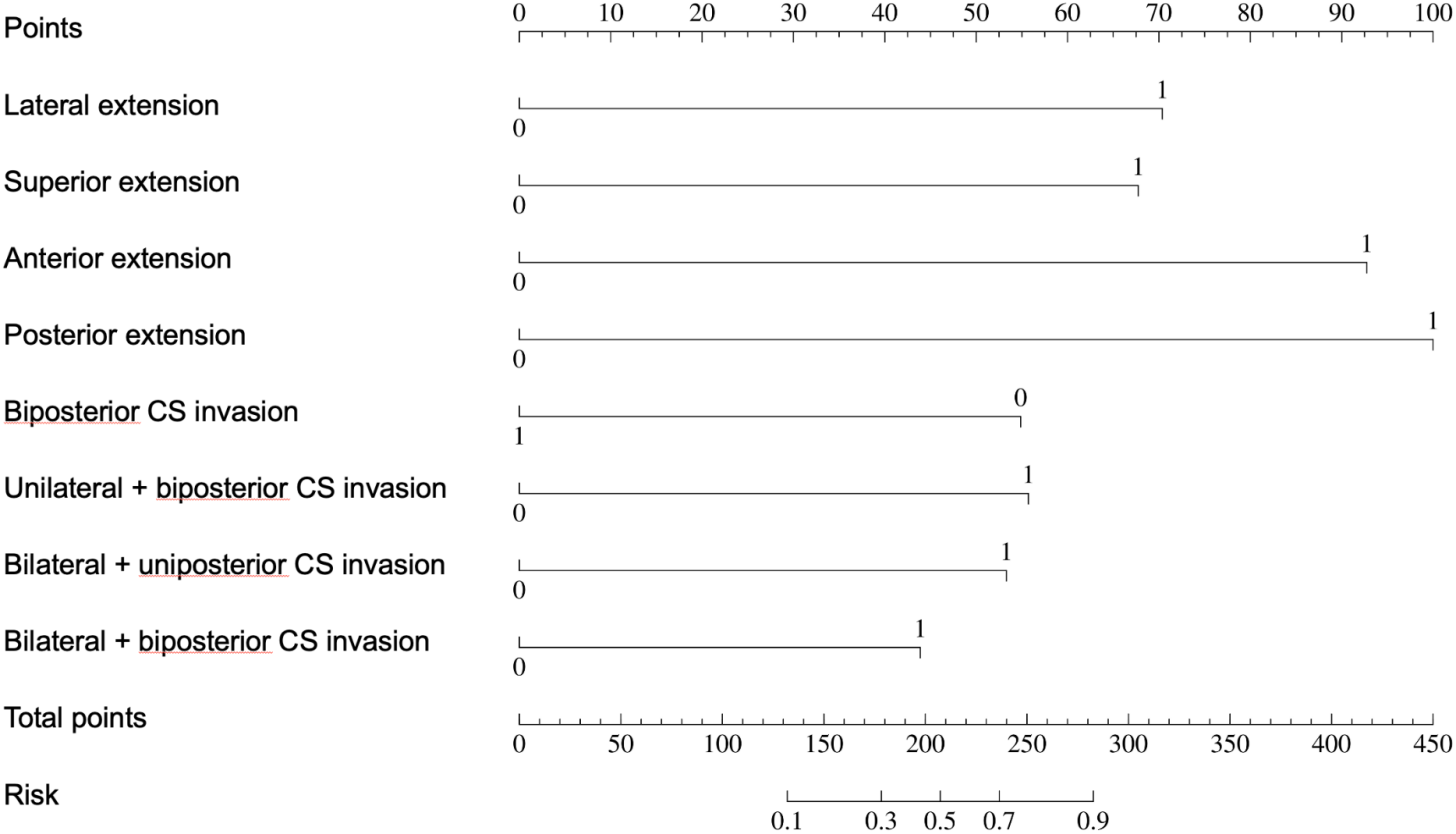
Nomogram for predicting near-total tesection (NTR) of pituitary adenomas. The nomogram assigns points based on the presence of various suprasellar extensions and types of cavernous sinus (CS) invasion. The total points for an individual patient are calculated by summing the points for each present feature. The total points are then used to determine the risk of NTR, with a higher total score indicating a greater likelihood of NTR.

Univariate regression analyses were conducted first. The suprasellar lateral extension beyond the defined range was significantly associated with a higher risk of near-total resection (NTR), with an odds ratio (OR) of 18.14 (95% CI: 10.96 - 30.03, P < 0.05). Similarly, the suprasellar superior extension beyond the range also showed a significant association with an increased risk of NTR, with an OR of 29.78 (95% CI: 17.35-51.12, P < 0.05). Anterior extension (A > C) and posterior extension (P > V) were similarly associated with higher risks of NTR, with ORs of 17.87 (95% CI: 10.13-31.52, P < 0.05) and 14.06 (95% CI: 7.49-26.39, P < 0.05), respectively. For CS invasion, except for uniposterior and unilateral + uniposterior CS invasions where no statistical significance was observed, all other cases showed a significant association with an increased risk of NTR (**Table 4**). Given the multitude of variables involved and the strong collinearity among them, LASSO regression was used for screening to identify potential predictive factors, ensuring avoidance of overfitting and enhancement of the model’s robustness. The results identified 8 anatomical landmarks (**Table 5**, **Figure 3**, **Supplementary Figure 1**). Multivariate regression analysis based on anatomical landmarks selected by LASSO regression revealed significant predictors of NTR. For suprasellar extension, lateral extension was significantly associated with an increased risk of NTR, with an odds ratio (OR) of 7.79 (95% CI: 3.07-19.77, P < 0.05). Similarly, superior extension, with an OR of 7.21 (95% CI: 2.81-18.51, P < 0.05), anterior extension, with an OR of 14.97 (95% CI: 5.19-43.17, P < 0.05), posterior extension, with an OR of 18.50 (95% CI: 4.80-71.23, P < 0.05) were significantly associated with an increased risk of NTR. Regarding CS invasion, unilateral + biposterior CS invasion, with an OR of 5.08 (95% CI: 1.61-16.02, P < 0.05) and bilateral + uniposterior CS invasion, with an OR of 4.74 (95% CI: 1.29-17.45, P < 0.05), showed a significant association with NTR. whereas biposterior, bilateral + biposterior CS invasion was not significantly associated with NTR (**Table 6**, **Figure 4**). According to the nomogram, posterior extension was identified as the strongest predictor of NTR, followed by anterior, lateral, and superior extensions. CS invasion, including biposterior, unilateral + biposterior, and bilateral + uniposterior invasions, were significantly associated with an increased risk of NTR (**Figure 5**).

**Table 4:** Univariate logistic regression based on anatomical landmarks and EOR.

Anatomical Landmarks		*P*	OR (95%CI)
Suprasellar	Lateral extension $\left(\left|k_{1}|>|k_{2}\right|\right)$	<.001*	18.14 (10.96 ~ 30.03)
	Superior extension $\left(\left|k_{3}|>|k_{4}\right|\right)$	<.001*	29.78 (17.35 ~ 51.12)
	Anterior extension (A > C)	<.001*	17.87 (10.13 ~ 31.52)
	Posterior extension (P > V)	<.001*	14.06 (7.49 ~ 26.39)
CS	Unilateral	0.003*	0.05 (0.01 ~ 0.36)
	Bilateral	<.001*	0.14 (0.05 ~ 0.40)
	Uniposterior	0.071	0.49 (0.23 ~ 1.06)
	Biposterior	0.002*	0.15 (0.05 ~ 0.49)
	Unilateral + uniposterior	0.191	0.61 (0.29 ~ 1.28)
	Unilateral + biposterior	<.001*	9.93 (5.12 ~ 19.28)
	Bilateral + uniposterior	<.001*	9.76 (4.84 ~ 19.65)
	Bilateral + biposterior	<.001*	57.21 (16.98 ~ 192.75)

CS, Cavernous sinus; OR, Odds ratio; CI, Confidence interval. The bold values and the symbol * indicate that the results are statistically significant.

**Table 5:** Lasso regression results for anatomical landmarks.

Anatomical Landmarks		Coefficient	*P*
Suprasellar	Lateral extension $\left(\left|k_{1}|>|k_{2}\right|\right)$	0.62	<0.05*
	Superior extension $\left(\left|k_{3}|>|k_{4}\right|\right)$	0.87	<0.05*
	Anterior extension (A > C)	0.71	<0.05*
	Posterior extension (P > V)	0.46	<0.05*
CS	Unilateral	0.00	<0.05*
	Bilateral	0.00	<0.05*
	Uniposterior	0.00	<0.05*
	Biposterior	-0.44	<0.05*
	Unilateral + uniposterior	0.00	0.37
	Unilateral + biposterior	0.29	<0.05*
	Bilateral + uniposterior	0.22	<0.05*
	Bilateral + biposterior	0.17	<0.05*

CS, Cavernous sinus.The bold values and the symbol * indicate that the results are statistically significant.

**Table 6:** Multivariate regression based on anatomical landmarks selected by lasso regression.

Anatomical Landmarks		*P*	OR (95%CI)
Suprasellar	Lateral extension $\left(\left|k_{1}|>|k_{2}\right|\right)$	<.001*	7.79 (3.07 ~ 19.77)
	Superior extension $\left(\left|k_{3}|>|k_{4}\right|\right)$	<.001*	7.21 (2.81 ~ 18.51)
	Anterior extension (P > C)	<.001*	14.97 (5.19 ~ 43.17)
	Posterior extension (P > V)	<.001*	18.50 (4.80 ~ 71.23)
CS	Biposterior	0.095	0.20 (0.03 ~ 1.32)
	Unilateral + biposterior	0.006*	5.08 (1.61 ~ 16.02)
	Bilateral + uniposterior	0.019*	4.74 (1.29 ~ 17.45)
	Bilateral + biposterior	0.261	3.60 (0.39 ~ 33.53)

CS, Cavernous sinus; OR, Odds ratio; CI, Confidence interval.The bold values and the symbol * indicate that the results are statistically significant.

### 3.4 The clinical prediction model based on anatomical landmarks

**Figure 6:**
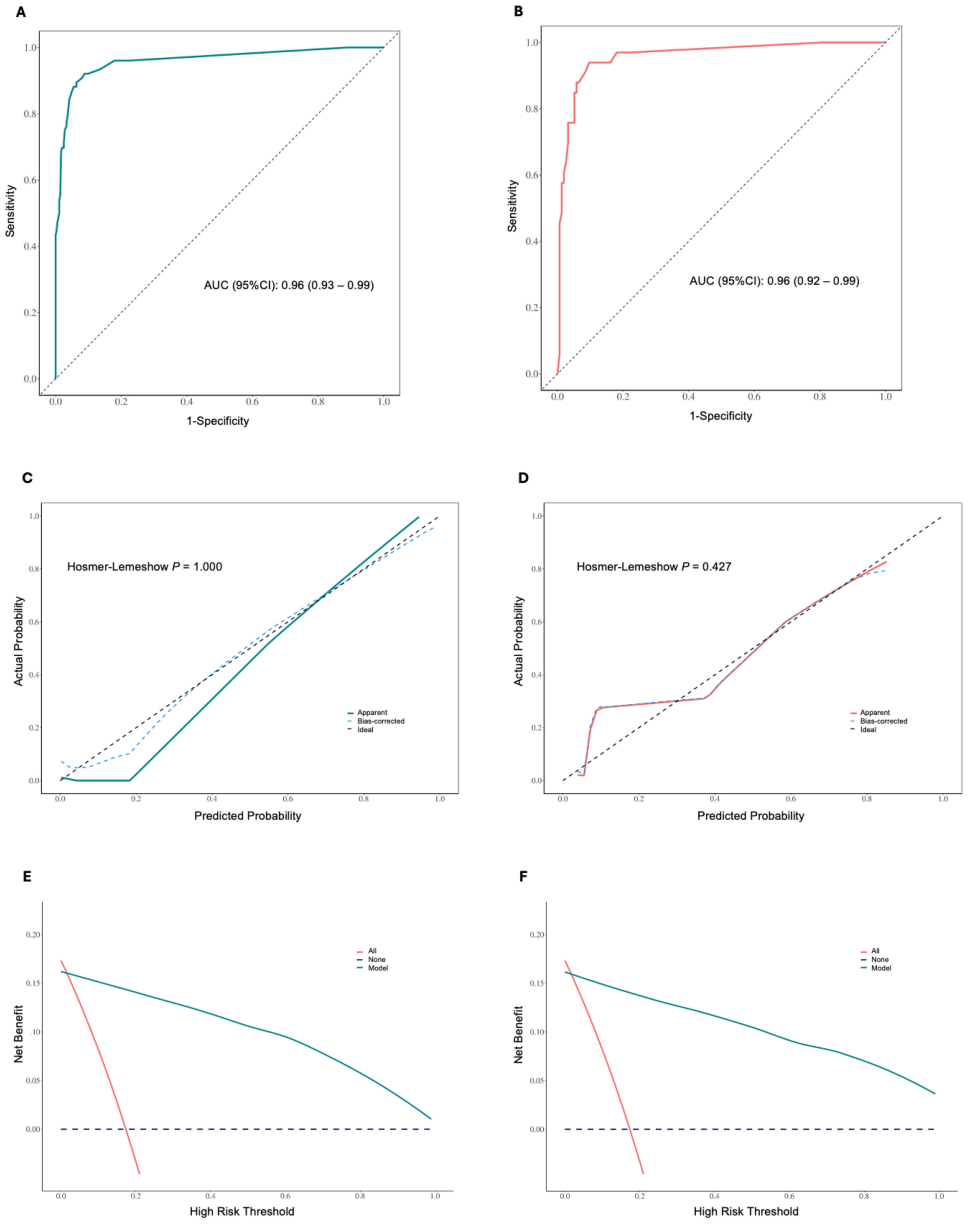
**(A)** Receiver operating characteristic (ROC) curve for the training set, showing an area under the curve (AUC) of 0.96 (95% CI: 0.93 – 0.99). **(B)** ROC curve for the validation set, showing an AUC of 0.96 (95% CI: 0.92 – 0.99). **(C)** Calibration curve for the training set, showing apparent, bias-corrected, and ideal calibration. The Hosmer-Lemeshow test indicates a good fit (P = 1.000). **(D)** Calibration curve for the validation set, showing apparent, bias-corrected, and ideal calibration. The Hosmer-Lemeshow test indicates a good fit (P = 0.427). The closer the lines are to the diagonal dashed line, the better the model’s predictions align with the actual outcomes. **(E)** Decision curve analysis (DCA) in the training set. **(F)** DCA in the validation set. The y-axis represents the net benefit, and the x-axis represents the high-risk threshold. The green line indicates the net benefit of the nomogram model, the red line represents the assumption that all patients experience the event, and the blue dashed line represents the assumption that no patients experience the event. The model provides a net benefit across a range of high-risk thresholds.

Patients were randomly assigned to training and validation sets in a 7:3 ratio (**Supplementary Tables 1**, **2**). Based on the regression analysis, a Receiver Operating Characteristic (ROC) curve was plotted to evaluate the model’s performance at different decision thresholds. In the training set, the ROC curve showed an AUC of 0.96 (95% CI: 0.93-0.99) (**Figure 6A**), and in the validation set, the AUC was 0.96 (95% CI: 0.92-0.99) (**Figure 6B**). The calibration curves for both the training and validation sets showed no significant deviation between the predicted and actual probabilities, as indicated by a Hosmer-Lemeshow test with P = 1.00 (**Figure 6C**) and P = 0.427 (**Figure 6D**), respectively. The apparent and bias-corrected lines closely followed the ideal line, suggesting a high degree of agreement between predictions and actual outcomes. In the decision curve analysis (DCA) of the training (**Figure 6E**) and validation sets (**Figure 6F**), the green line represents the prediction model, the red line represents the assumption that all patients experience the event, and the blue dashed line represents the assumption that no patients experience the event. The green line is above the red and blue lines across a range of high-risk thresholds from approximately 0.1 to 0.7, indicating that the prediction model provides a net benefit in this range. This suggests that using the model to predict the EOR of EEA can improve decision-making compared to assuming all or no patients will experience the event within this threshold range.

### 3.5 Survival analysis based on anatomical landmarks

**Figure 7:**
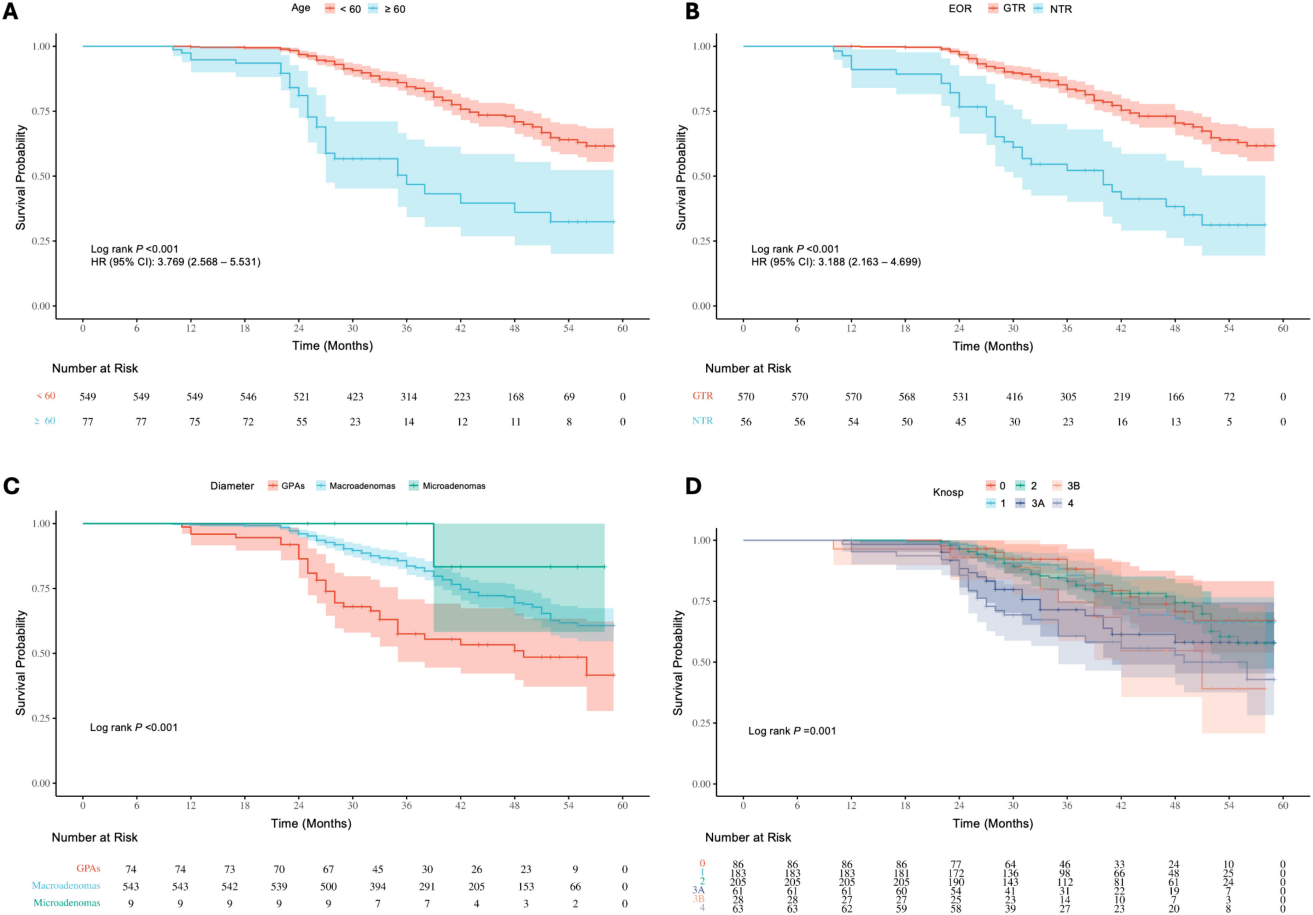
**(A)** Kaplan-Meier survival curves for recurrence/progression stratified by age (< 60 years *vs*. ≥ 60 years). **(B)** Kaplan-Meier survival curves stratified by extent of resection (EOR), comparing GTR and NTR groups. **(C)** Kaplan-Meier survival curves stratified by tumor diameter, showing differences among GPAs, macroadenomas, and microadenomas. **(D)** Kaplan-Meier survival curves stratified by Knosp grade.

**Figure 8:**
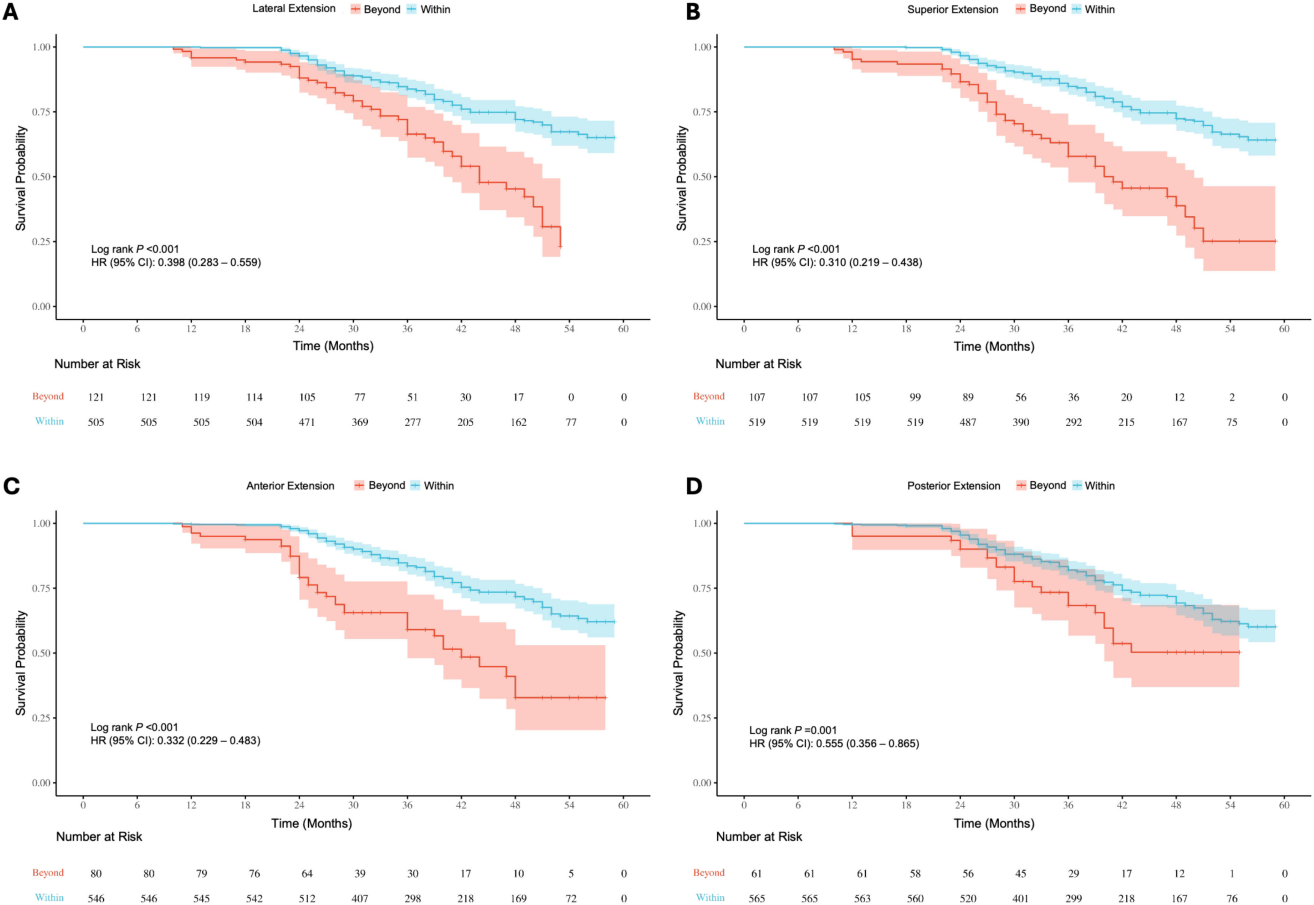
Kaplan-Meier survival curves for recurrence/progression stratified by lateral **(A)**, superior **(B)**, anterior **(C)** and posterior **(D)** extension in the suprasellar region, comparing patients with tumors extending beyond anatomical landmarks to those within the landmarks.

**Figure 9:**
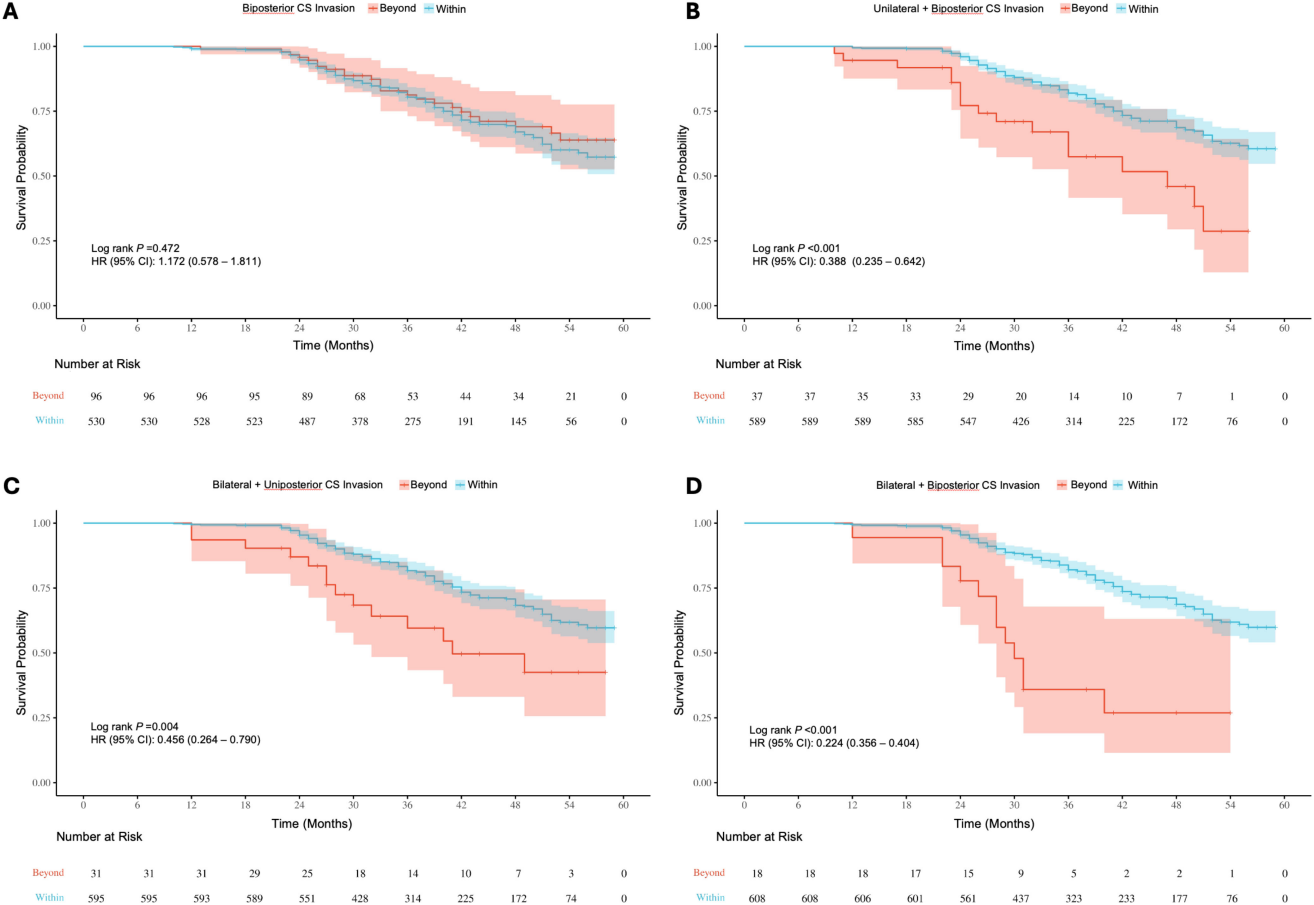
**(A)** Kaplan-Meier survival curves for recurrence/progression stratified by biposterior **(A)**, unilateral + biposterior **(B)**, bilateral + uniposterior **(C)** and bilateral + biposterior **(D)** cavernous sinus (CS) invasion, comparing patients with or without corresponding CS invasion.

Survival analysis of baseline information showed significant differences (P < 0.05) in the risk of recurrence/progression across various groups stratified by patient age (HR: 3.769, 95% CI: 2.568-5.531) (**Figure 7A**), EOR (HR: 3.188, 95% CI: 2.163-4.699) (**Figure 7B**), PAs diameter (**Figure 7C**), and Knosp grade (**Figure 7D**). No statistical significance was observed on the Kaplan-Meier Survival Curves between the pathological types of PAs and the groups with Ki-67 < 3% and ≥ 3% (**Supplementary Figure 2**). PAs with suprasellar extension beyond anatomical landmarks also showed a higher probability of recurrence/progression (P < 0.05), for lateral (HR: 2.514, 95% CI: 1.789-3.535) (**Figure 8A**), superior (HR: 3.228, 95% CI: 2.284-4.563) (**Figure 8B**), anterior (HR: 3.008, 95% CI: 2.070-4.370) (**Figure 8C**), and posterior extensions (HR: 1.801, 95% CI: 1.156-2.807) (**Figure 8D**). For CS invasion, no significant difference in recurrence/progression was observed between the groups with and without biposterior CS invasion (**Figure 9A**). However, the presence of unilateral + biposterior (HR: 2.576, 95% CI: 1.557-4.264) (**Figure 9B**), bilateral + uniposterior (HR: 2.192, 95% CI: 1.266-3.795) (**Figure 9C**), and bilateral + biposterior CS invasions (HR: 4.468, 95% CI: 2.472-8.074) (**Figure 9D**) was significantly associated with a higher probability of recurrence/progression (P < 0.05).

## 4 Discussion

This study comprehensively analyzed the EOR and its influencing factors in patients undergoing EEA for PAs. We identified significant associations between various anatomical landmarks and surgical outcomes. Incorporating LASSO regression allowed us to screen potential predictive factors effectively, reducing the risk of overfitting and enhancing the model’s robustness. The selected anatomical landmarks were: lateral, superior, anterior, and posterior suprasellar extension, as well as biposterior, unilateral + biposterior, bilateral + uniposterior, and bilateral + biposterior CS invasion. The resulting clinical prediction model demonstrated excellent performance, as evidenced by high AUC values in both training and validation sets. The calibration curves indicated a high degree of agreement between predicted and actual outcomes, while decision curve analysis highlighted the model’s net benefit across a range of risk thresholds. Survival analysis further confirmed significant differences in recurrence/progression risks based on patient age, PAs diameter, Knosp grade, and EOR. Additionally, suprasellar extensions beyond anatomical landmarks and specific patterns of CS invasion were strongly associated with higher probabilities of recurrence/progression. These findings underscore the critical role of detailed anatomical assessment and tailored surgical strategies in improving patient outcomes.

Among the gross classification systems for PAs, the Hardy classification assesses the extension of PAs in the sellar region indirectly through the morphology of the sella turcica on pneumoencephalography, it was historically significant as it first proposed considering the extension of PAs in the sellar region (18, 19).With the advent of CT and direct visualization of adenomas during surgery, Wilson et al. enhanced this classification by describing the lateral extension of PAs in the sella turcica (20). The Hardy-Wilson classification was commonly used until MRI became the standard for assessing sellar region lesions (21). The Knosp classification is now the most widely used and practical grading system for evaluating adenoma growth invading the cavernous sinus. Knosp et al. assess the degree of invasion based on preoperative MRI, focusing on the tumor’s position relative to the ICA (22). However, single coronal MRI images can be limited in accurately determining true invasion, and criteria based solely on the ICA line are insufficient as reliable indicators of CS invasion. Edal et al. introduced the SIPAP classification, emphasizing the extension of PAs outside the sellar region and their impact on adjacent structures (23, 24). While the combination of SIPAP grading and tumor size is significant for treatment and follow-up, differentiating postoperative residual adenomas from postoperative lesions can be challenging. Several studies have attempted to combine the Knosp-Hardy-Wilson classifications to describe tumor growth in all directions more accurately (21).

Despite the advancements in imaging techniques and the introduction of multiple classification systems combining tumor size, extension, and CS invasion, these classifications still have limitations. Most current systems are extensions or modifications of the foundational ideas proposed by KNOSP, HARDY, and WILSON (25–31). With the widespread use of endoscopy, these classification methods have room for further improvement.

Our study addressed several limitations of existing systems. Firstly, we included all sizes of pituitary adenomas in our analysis, unlike most previous studies that focused solely on GPAs. We observed that even smaller adenomas with significant skull base extension or cavernous sinus invasion can pose substantial surgical challenges. Secondly, we confined the surgical approach to the EEA. Thirdly, our evaluation system is adaptive because the anatomical landmarks we define are not entirely mechanical or fixed; they consider the displacement of normal anatomical structures caused by the tumor. For example, the anatomical landmark defined for anterior suprasellar extension is essentially the boundary between the PAs and normal brain tissue, which changes as the tumor grows. Similarly, in cases of posterior, lateral, and superior suprasellar extensions, the same principle applies. The relative position of the PAs to the anatomical landmarks, such as the clinoid and cavernous segments of the ICA, the anterior bend of the ICA, the clivus, and the sphenoid sinus, varies with tumor growth and is different for each patient. For assessing CS invasion, we referenced Fernandez-Miranda’s endoscopy-based CS segmentation, emphasizing the lateral and posterior CS regions relative to the ICA. These two regions are areas that are difficult for endoscopic instruments to safely reach during EEA surgery without causing damage to the associated blood vessels and nerves. Additionally, due to the tumor’s displacement, where tumor-induced shifts can create new surgical corridors by widening originally narrow spaces, and the width of the surgical pathways on both sides may be inconsistent. Therefore, we conducted a side-by-side discussion, which also reflects our adaptive approach. Lastly, considering the “bucket effect” in surgery, where the presence of a single factor can significantly impact surgical outcomes, we did not define or calculate a specific, mechanical “scoring system” or “cutoff value” to predict the probability of achieving total resection.

In summary, our proposed classification system is individualized and adaptive, focusing on the practicality of the EEA procedure. It considers the operational angles and reachability of endoscopic instruments, as well as anatomical variations and pathological displacements under surgical conditions. This innovation marks a significant improvement over existing classification systems.

### 4.1 Limitations

We used the cavernous sinus segmentation proposed by Juan C. Fernandez-Miranda and focused on the lateral and posterior compartments of the CS, considering the endoscopic surgery’s attack angle. However, the relatively low number of cases with bilateral + biposterior CS invasions limits the reliability of our statistical results. Future research should be focused on building upon the existing single-center retrospective study to further refine, optimize, and expand the applicability of this evaluation system through multi-center, prospective studies. In clinical practice, we have observed that even in cases where the tumor extends beyond the anatomical landmarks, GTR can still be achieved. This may be related to the tumor’s texture, the degree of vascular adhesion, or techniques such as removing the lower part of the tumor, causing the upper part to collapse, or using a combined surgical approach. External validation is necessary for future research, but it should be implemented after determining whether subtle technical and habitual differences in endoscopic surgery across different surgical teams and centers will affect the application of this evaluation system.

## 5 Conclusion

This study analyzed the EOR and its influencing factors in patients with PAs undergoing the EEA. Significant associations were found between anatomical landmarks and surgical outcomes. Key landmarks included suprasellar extensions in various directions and different patterns of CS invasion. The study demonstrated strong performance and robustness through regression analysis and clinical prediction modeling. Survival analysis confirmed higher recurrence/progression risks associated with specific landmarks and CS invasion patterns. This adaptive and individualized classification system improves surgical outcomes by considering operational angles, instrument reachability, and anatomical variations.

## Data Availability

The raw data supporting the conclusions of this article will be made available by the authors, without undue reservation.
