# Supplementary material for: Adaptive evaluation of gross total resection rates for endoscopic endonasal approach based on preoperative MRI morphological features of pituitary adenomas
Source: Front Oncol. 2024 Dec 17;14:1481899. doi: 10.3389/fonc.2024.1481899 (PMC11685135; doi:10.3389/fonc.2024.1481899)
Supplement: Supplementary file 1 [file DataSheet1.docx]

Supplementary Material

# Supplementary Figures and Tables

## Supplementary Figures


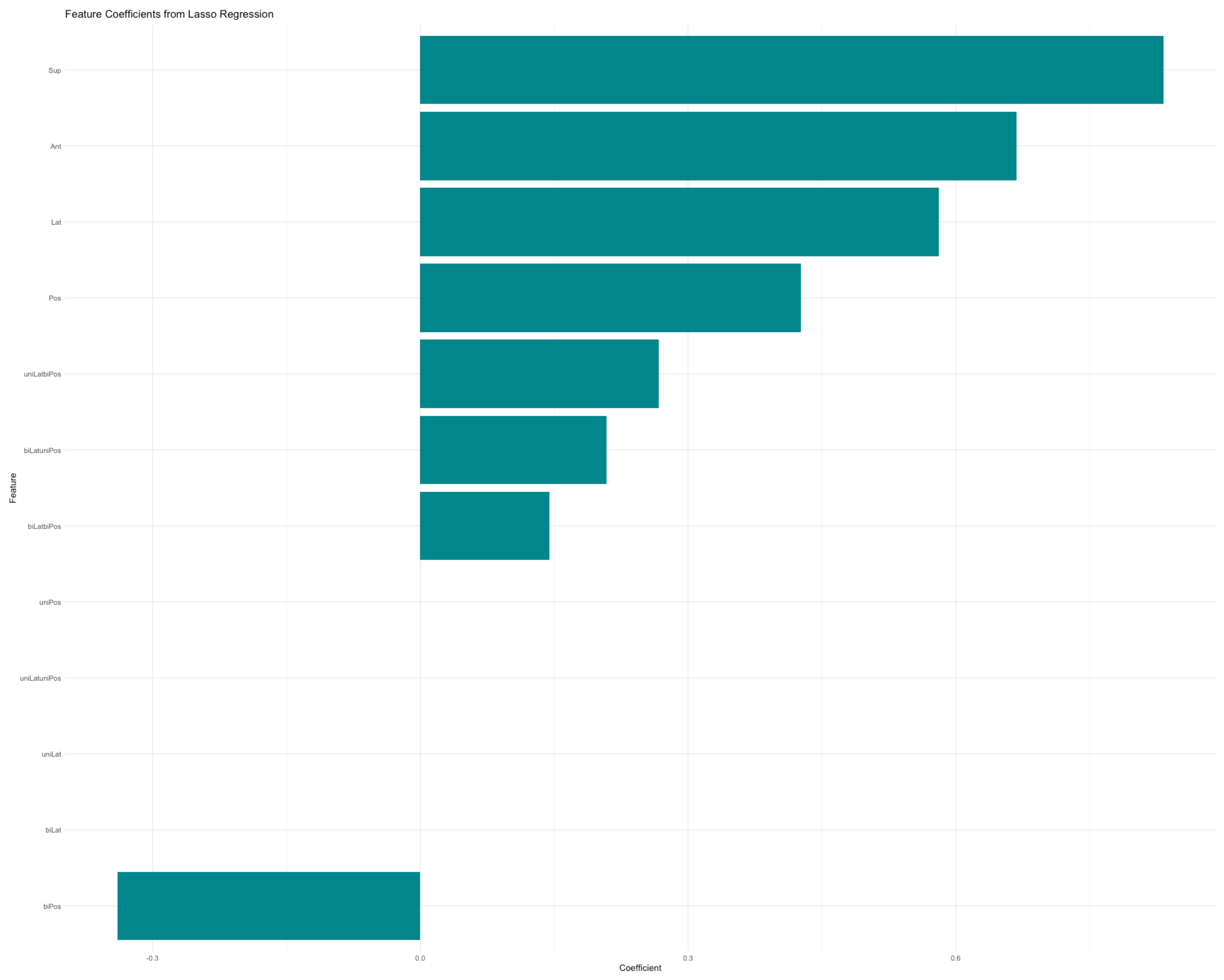


**Supplementary Figure 1.** Feature Coefficients from Lasso Regression. This bar plot displays the coefficients of the selected features from the Lasso regression model. The features include: superior extension (Sup), anterior extension (Ant), lateral extension (Lat), posterior extension (Pos), unilateral + biposterior cavernous sinus invasion (uniLatBiPos), bilateral + uniposterior cavernous sinus invasion (biLatUniPos), bilateral + biposterior cavernous sinus invasion (biLatBiPos), unilateral uniposterior cavernous sinus invasion (uniPos), and biposterior cavernous sinus invasion (BiPos). The coefficients indicate the strength and direction of the association of each feature with the outcome variable.

**
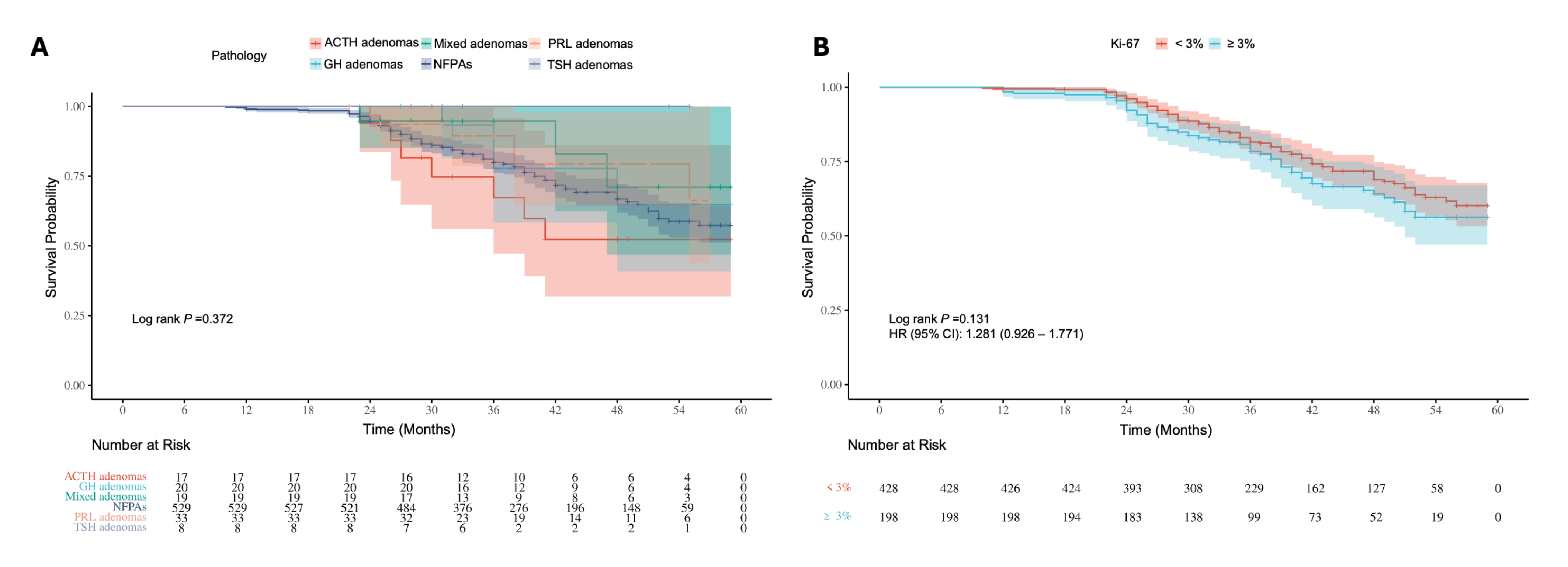
**

**Supplementary Figure 2.** A: Kaplan-Meier survival curves for recurrence/progression stratified by different pathological types of pituitary adenomas, including ACTH adenomas, GH adenomas, mixed adenomas, NFPAs, PRL adenomas, and TSH adenomas. B: Kaplan-Meier survival curves for recurrence/progression stratified by Ki-67 index, comparing patients with Ki-67 < 3% to those with Ki-67 ≥ 3%.

## Supplementary Tables

**Supplementary Table 1** Balance Test for Training and Validation Sets

| **Anatomical Landmarks，n (%)** | | **Total (626, 100)** | **Validation (188, 30.03)** | **Training (438, 69.97)** | ***P*** |
| --- | --- | --- | --- | --- | --- |
| Suprasellar | Lateral extension，n (%) | | | | 0.989 |
|  | Within | 503 (80.35) | 151 (80.32) | 352 (80.37) |  |
|  | Beyond (${\vert k}_{1}\vert>{\vert k}_{2}\vert$) | 123 (19.65) | 37 (19.68) | 86 (19.63) |  |
|  | Superior extension，n (%)22 | | | | 0.764 |
|  | Within | 504 (80.51) | 150 (79.79) | 354 (80.82) |  |
|  | Beyond (${\vert k}_{3}\vert>{\vert k}_{4}\vert$) | 122 (19.49) | 38 (20.21) | 84 (19.18) |  |
|  | Anterior extension, n (%) | | | | 0.229 |
|  | Within | 551 (88.02) | 161 (85.64) | 390 (89.04) |  |
|  | Beyond (A$>$ C) | 75 (11.98) | 27 (14.36) | 48 (10.96) |  |
|  | Posterior extension, n (%) | | | | 0.491 |
|  | Within | 572 (91.37) | 174 (92.55) | 398 (90.87) |  |
|  | Beyond (P$>$ V) | 54 (8.63) | 14 (7.45) | 40 (9.13) |  |
| CS | Biposterior, n (%) | | | | 0.517 |
|  | Non-invasion | 534 (85.30) | 163 (86.70) | 371 (84.70) |  |
|  | Invasion | 92 (14.70) | 25 (13.30) | 67 (15.30) |  |
|  | Unilateral + biposterior，n (%) | | | | 0.177 |
|  | Non-invasion | 583 (93.13) | 179 (95.21) | 404 (92.24) |  |
|  | Invasion | 76 (12.14) | 9 (4.79) | 34 (7.76) |  |
|  | Bilateral + uniposterior，n (%) | | | | 0.236 |
|  | Non-invasion | 587 (93.77) | 173 (92.02) | 414 (94.52) |  |
|  | Invasion | 39 (6.23) | 15 (7.98) | 24 (5.48) |  |
|  | Bilateral + biposterior, n (%) | | | | 0.599 |
|  | Non-invasion | 595 (95.05) | 180 (95.74) | 415 (94.75) |  |
|  | Invasion | 31 (4.95) | 8 (4.26) | 23 (5.25) |  |

**Supplementary Table 2** Anatomical Landmarks and Surgery Outcomes by EOR in Training Group

| **Anatomical Landmarks，n (%)** | | **Total (438, 100)** | **GTR (362, 82.65)** | **NTR (76, 17.35)** | ***P*** |
| --- | --- | --- | --- | --- | --- |
| Suprasellar | Lateral extension，n (%) | | | | **<0.001******* |
|  | Within | 351 (80.14) | 328 (93.45) | 23 (6.55) |  |
|  | Beyond (${\vert k}_{1}\vert>{\vert k}_{2}\vert$) | 87 (19.86) | 34 (39.08) | 53 (60.92) |  |
|  | Superior extension，n (%)22 | | | | **<0.001*** |
|  | Within | 351 (80.14) | 324 (92.31) | 27 (7.69) |  |
|  | Beyond (${\vert k}_{3}\vert>{\vert k}_{4}\vert$) | 87 (19.86) | 37 (42.53) | 50 (57.47) |  |
|  | Anterior extension, n (%) | | | | **<0.001*** |
|  | Within | 382 (87.21) | 345 (98.01) | 37 (9.69) |  |
|  | Beyond (A$>$ C) | 56 (12.79) | 17 (30.36) | 39 (69.64) |  |
|  | Posterior extension, n (%) | | | | **<0.001*** |
|  | Within | 409 (93.38) | 353 (86.31) | 56 (13.69) |  |
|  | Beyond (P$>$ V) | 29 (6.62) | 9 (31.03) | 20 (68.97) |  |
| CS | Biposterior, n (%) | | | | **<0.001*** |
|  | Non-invasion | 381 (86.99) | 308 (80.84) | 73 (19.16) |  |
|  | Invasion | 57 (13.01) | 54 (94.74) | 3 (5.26) |  |
|  | Unilateral + biposterior，n (%) | | | | **<0.001*** |
|  | Non-invasion | 404 (92.24) | 351 (86.88) | 53 (13.12) |  |
|  | Invasion | 34 (7.76) | 11 (32.35) | 23 (67.65) |  |
|  | Bilateral + uniposterior，n (%) | | | | **<0.001*** |
|  | Non-invasion | 414 (94.52) | 353 (85.27) | 61 (14.73) |  |
|  | Invasion | 24 (5.48) | 9 (37.50) | 15 (62.50) |  |
|  | Bilateral + biposterior, n (%) | | | | **<0.001*** |
|  | Non-invasion | 417 (95.21) | 360 (86.33) | 57 (13.67) |  |
|  | Invasion | 21 (4.79) | 2 (9.52) | 19 (90.48) |  |

CS: Cavernous sinus, GTR: Gross total resection, NTR: Near total resection.
